# Open-Space Vehicle Positioning Algorithm Based on Detection and Tracking in Video Sequences

**DOI:** 10.3390/s22239098

**Published:** 2022-11-23

**Authors:** Zhaoyu Zheng, Kai Zhang

**Affiliations:** 1 Tsinghua Shenzhen International Graduate School, Tsinghua University, Shenzhen 518055, China; 2 Research Institute of Tsinghua, Pearl River Delta, Guangzhou 510530, China

**Keywords:** on-street parking, parallel detection, geometric matching, vehicle tracking

## Abstract

In on-street parking lots, it is very important to obtain the positions and license plate numbers of the vehicles for charging and management. Existing algorithms usually detect vehicles from an input image first, then localize their license plates, and finally recognize the license plate numbers. However, they are of high time and space complexity and cannot work if the vehicles or license plates are obscured. Therefore, this paper proposes an open-space vehicle positioning algorithm based on detection and tracking in video sequences. The work is as follows: (1) To reduce the time and space complexity, parallel detection of vehicles and license plates is carried out. Then, geometric matching is performed to accomplish the correspondences between them. (2) To track vehicles and license plates, this paper improves DeepSORT by combining with integrated voting based on the historical license plate library. (3) To accurately judge the vehicle behavior of entry or exit, a cumulative state detector is designed to increase the fault-tolerance of the proposed algorithm. The experimental results reveal that the proposed algorithm makes improvements in model parameters, inference speed, and tracking accuracy, demonstrating that it can be well applied to open-space vehicle positioning.

## 1. Introduction

**Figure 1:**
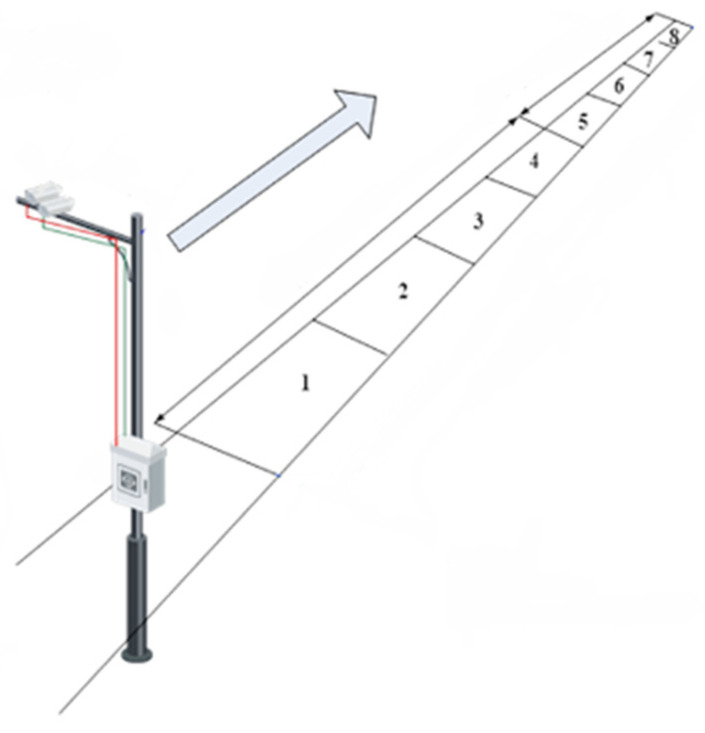
The method of camera installation.

In the past ten years, the number of vehicles in China has increased from 225 million to 395 million [1,2], leading to the shortage of parking spaces. Consequently, many cities have embarked on building on-street parking lots, which can be put into use at the least cost by simply painting parking lines on the roads. However, due to the uncertainty of vehicle entry and exit directions, it causes difficulties in managing the on-street parking lots. Currently, on-street parking management mainly relies on regular patrols by staff, resulting in a large amount of wasted labor. Therefore, it is significant to design a vehicle positioning algorithm applicable to open space. A feasible approach is to install cameras on high places next to the on-street parking lots to obtain information of vehicles through perception algorithms, as shown in Figure 1. Under the circumstance, each camera monitors multiple parking spaces.

In order to facilitate the management of vehicles, the required vehicle information includes positions of vehicles, license plate numbers, and the correspondences between both. RCNN series methods [3,4,5,6,7] and YOLO series methods [8,9,10,11,12,13,14] can be used to locate the vehicles and the license plates. In addition, LPRNet [15] could be applied to recognize the license plate numbers after extracting the license plates. In terms of the correspondences between the vehicles and their license plate numbers, VT-LPR [16] first located the vehicles from an input image, then detected their license plates from the extracted vehicles, and finally recognized the license plate numbers, at which time the vehicles and their license plate numbers were in mutual correspondences.

However, this method takes 95.3 ms to process an image and its model parameter is up to 14,025,644 (see Section 4.1 for details). Moreover, at certain angles, the vehicles and license plates may be obscured, causing objects to be invisible. Therefore, it is necessary to speed up the inference, reduce model parameters, and track the vehicles and their license plate numbers.

Object tracking algorithms [17,18,19,20,21] can track the target vehicles and correlate them with the previous trajectories even if the targets reappear after being obscured. It is worth noting that they only use the positions and appearances of the vehicles for tracking without license plate numbers, which makes them invalid predictors of the invisible license plate numbers.

To address the above problems, this paper proposes an open-space vehicle positioning algorithm based on detection and tracking in video sequences. To speed up the inference and reduce model parameters, it carries out parallel detection of the vehicles and license plates, and then matches them based on their geometric relationships. To increase the accuracy of license plate number prediction, it improves DeepSORT [18] by combining with integrated voting. Even if a license plate is not visible in the current frame, its number can also be inferred from the historical license plate library, effectively solving the problem of invisibility at certain angles. Finally, the cumulative state detector is designed to judge the vehicle behavior of entry or exit, which reduces the impact of data perturbation, thereby increasing the fault-tolerance of the proposed algorithm.

As none of the existing datasets are specifically used for on-street parking scenes, to better evaluate the performance of the proposed algorithm, the data of on-street parking vehicles are collected and annotated to form three datasets, including a vehicle detection dataset, a vehicle tracking dataset, and a vehicle entry–exit dataset. The results of the algorithm in these datasets are as follows: 98.1% for mean average precision at Intersection-over-Union(IoU) = 0.5, 92.1% for multi-object tracking accuracy, 89.4% for multi-object tracking precision, and 92.95% for vehicle entry–exit license plate number accuracy, which reflects the excellent achievements of the algorithm.

The contributions are as follows:

(1) Carry out parallel detection of vehicles and license plates, and then match them based on their geometric relationships. Compared with locating the vehicles first and then the license plates, this algorithm has only half the number of model parameters and its inference speed is nearly four times faster.

(2) Improve DeepSORT by combining with integrated voting to infer vehicles’ positions and license plate numbers. Compared with DeepSORT, the algorithm has a 42.31 percentage point increase in vehicle entry–exit license plate number accuracy.

(3) Judge the vehicle behavior of entry or exit by the cumulative state detector to increase the fault-tolerance. Compared with the adjacent state detector, the algorithm makes considerable improvement in the precision of the entry and exit time.

The rest of this paper is organized as follows. It first discusses the materials and methods in Section 2. Section 3 introduces the datasets and Section 4 presents experimental results and analysis. Section 5 showcases the limitations of the proposed algorithm, and the last part shows the conclusions and future work.

## 2. Materials and Methods

### 2.1. Literature Review

Object detection algorithms are used to locate the vehicles and the license plates, which can be divided into two-stage and one-stage approaches. Two-stage approaches first generate possible regions and then obtain their classification and regression results. Region-based Convolutional Neural Network (R-CNN) series methods are typical two-stage detectors. R-CNN [3] introduced convolutional neural networks to object detection for the first time. Fast R-CNN [4] optimized the efficiency of bounding box regression and Faster R-CNN [5] reduced the time of possible region generation. Feature Pyramid Network (FPN) [6] and Cascade R-CNN [7] further improved the precision of Faster R-CNN by adding cross-layer connections and cascading multiple Faster R-CNN heads.

Compared to two-stage approaches, one-stage approaches regress the parameters of the bounding boxes directly, leading to a faster speed of inference. Redmon [8] et al. presented a real-time end-to-end detection algorithm You Only Look Once (YOLO), in which the grids divided from the image were responsible for detecting objects. YOLOv2 [9] optimized YOLO by anchor-based relative position prediction. YOLOv3 [10] further adopted multi-scale feature maps based on YOLOv2 to detect objects of different sizes. YOLOv4 [11] and YOLOv5 [12] integrated a variety of advanced algorithms on the basics of YOLOv3 to improve its precision while greatly reducing inference time and memory consumption. YOLOv6 [13] and YOLOv7 [14] introduced structural re-parameterization [22] to reduce model parameters and computation.

Object tracking algorithms can track the target vehicles and correlate them with the previous trajectories even if the targets reappear after being obscured. Bewley et al. [17] proposed Simple Online and Realtime Tracking (SORT), which included the motion estimation and the data association. The motion estimation utilized the Kalman filter [23] to predict the positions and sizes of bounding boxes, and the data association applied the Hungarian algorithm [24] to obtain the matching results. DeepSORT [18] adopted a wide residual network [25] to extract the appearance features of the targets and then combined them with motion information. Some approaches have been proposed to simplify the tracking process. The Jointly learns the Detector and Embedding model (JDE) [19] merged the appearance embedding model into the detector, CenterTrack [20] combined detection and tracking into a single network, and FairMOT [21] integrated the detection framework with ReID.

### 2.2. Framework Overview

**Figure 2:**
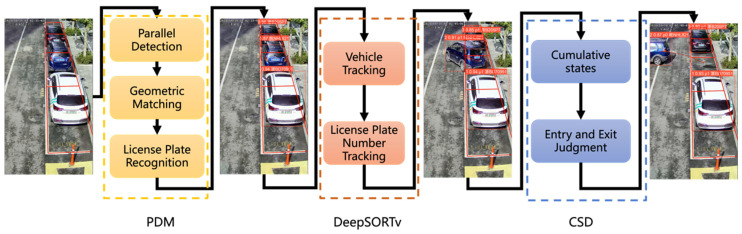
The overall framework of the proposed algorithm.

The overall framework of the proposed algorithm in this paper is presented in Figure 2. The whole framework is composed of three modules: Parallel Detection and Matching (PDM), DeepSORT with integrated voting (DeepSORTv), and Cumulative State Detector (CSD).

The PDM module is used to obtain vehicle information. The detailed proceedings are as follows: it first carries out parallel detection of the vehicles and the license plates, then matches them based on their geometric relationships, and finally recognizes the license plate numbers, to accomplish the correspondences between the vehicles and the license plate numbers.

The DeepSORTv module is designed to track the vehicles and license plate numbers. The vehicle tracking predicts the positions of the target vehicles by the Kalman Filter, and the tracking of the license plate numbers is achieved through integrated voting.

The CSD module is applied to judge the vehicle behavior of entry or exit. It identifies different vehicle states according to whether it is in the parking lot or not, and judges the vehicle behavior by its cumulative states.

### 2.3. Vehicle Information Acquisition

**Figure 3:**
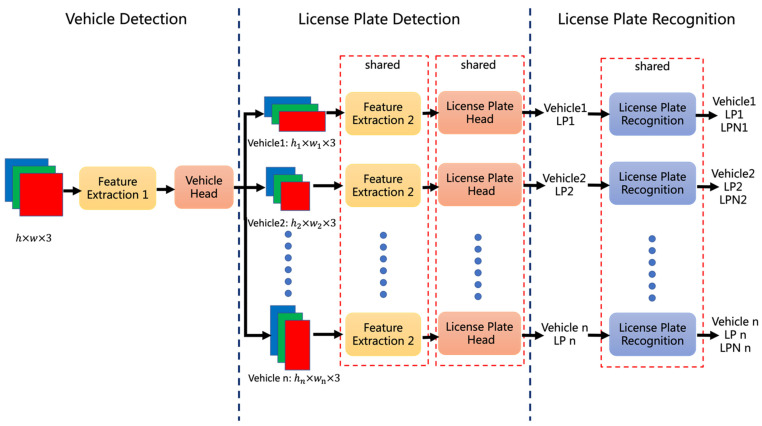
The structure of VLN.

Vehicle information requires not only the positions of vehicles and license plate numbers, but also the correspondences between both. Vehicle-License plate-Number (VLN), a conventional architecture, is shown in Figure 3, where license plate is abbreviated as lp, and license plate number is abbreviated as lpn. It first locates the vehicle in the image with a detector, then detects its license plate with another detector, and finally recognizes the license plate number. The advantage of this architecture is that the license plate number corresponds to that vehicle without specially designed matching.

However, there are two disadvantages of the above architecture: (1) High spatial complexity. It does not share the parameters of the feature extraction network, so it applies two different models for the detection of vehicles and their license plates, resulting in high spatial complexity. (2) High time complexity. It needs to detect all vehicles in the image first, then traverses each of them to detect the license plates in turn. In this scene, the required time for detection is positively correlated with the number of vehicles, so the time complexity is high if there is a large number of them.

**Figure 4:**
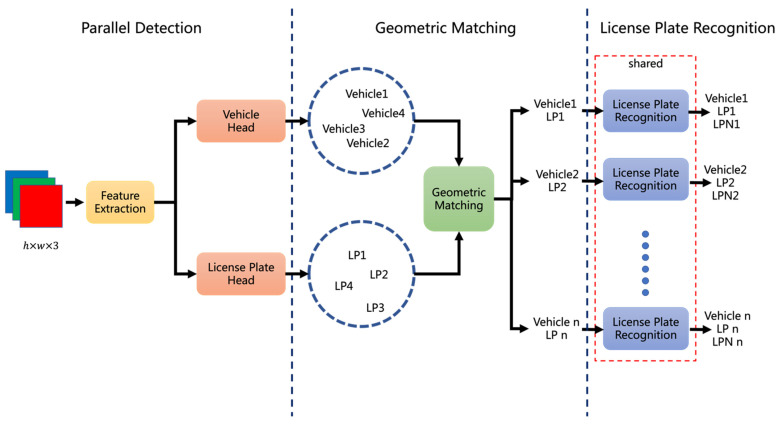
The structure of the proposed PDM.

To remove the above disadvantages, this paper designs a new method, Parallel Detection and Matching (PDM), as shown in Figure 4, in which YOLOv5 is utilized for feature extraction and LPRNet for license plate recognition. An image is fed into the shared feature extraction network, following with two detection heads, the vehicle head and the license plate head, to, respectively, obtain the positions of the vehicles and the license plates. Then, the geometric matching between the vehicles and the license plates is implemented to assign the corresponding license plate to each vehicle.

In order to better describe the matching process, this paper supposes that the vehicle bounding box is $v^{\left(i\right)}=\left(v_{x_{1}}^{\left(i\right)},v_{y_{1}}^{\left(i\right)},v_{x_{2}}^{\left(i\right)},v_{y_{2}}^{\left(i\right)}\right)\left(i=1,2,3,\ldots n\right)$ and the license plate bounding box is $lp^{\left(j\right)}=\left(lp_{x_{1}}^{\left(j\right)},lp_{y_{1}}^{\left(j\right)},lp_{x_{2}}^{\left(j\right)},lp_{y_{2}}^{\left(j\right)}\right)\left(j=1,2,3,\ldots m\right)$, where $\left(v_{x_{1}}^{\left(i\right)},v_{y_{1}}^{\left(i\right)}\right)$ and $\left(v_{x_{2}}^{\left(i\right)},v_{y_{2}}^{\left(i\right)}\right)$ are, respectively, the top-left and bottom-right corners of $v^{\left(i\right)}$; $\left(lp_{x_{1}}^{\left(j\right)},lp_{y_{1}}^{\left(j\right)}\right)$ and $\left(lp_{x_{2}}^{\left(j\right)},lp_{y_{2}}^{\left(j\right)}\right)$ are, respectively, the top-left and bottom-right corners of $lp^{\left(j\right)}$.

The steps of the geometric matching are as follows:

Step 1: Rough filtering. The license plates that are not in the lower half of the vehicle bounding box are filtered out, and the retained license plate bounding boxes need to satisfy Equations (1)–(4):

(1) $$lp_{x_{1}}^{\left(j\right)}>v_{x_{1}}^{\left(i\right)},$$

(2) $$lp_{y_{1}}^{\left(j\right)}>\left(v_{y_{1}}^{\left(i\right)}+v_{y_{2}}^{\left(i\right)}\right)/2,$$

(3) $$lp_{x_{2}}^{\left(j\right)}<v_{x_{2}}^{\left(i\right)},$$

(4) $$lp_{y_{2}}^{\left(j\right)}<v_{y_{2}}^{\left(i\right)}.$$

Step 2: Precise matching. The license plate nearest to the center point of the lower half of the vehicle bounding box is the final matching result, which is selected from the unfiltered license plate bounding boxes and calculated by Equations (5)–(9):

(5) $$v_{cx}^{\left(i\right)}=\frac{v_{x_{1}}^{\left(i\right)}+v_{x_{2}}^{\left(i\right)}}{2},$$

(6) $$v_{cy}^{\left(i\right)}=\frac{v_{y_{1}}^{\left(i\right)}+3\times v_{y_{2}}^{\left(i\right)}}{4},$$

(7) $$lp_{cx}^{\left(j\right)}=\frac{lp_{x_{1}}^{\left(j\right)}+lp_{x_{2}}^{\left(j\right)}}{2},$$

(8) $$lp_{cy}^{\left(j\right)}=\frac{lp_{y_{1}}^{\left(j\right)}+lp_{y_{2}}^{\left(j\right)}}{2},$$

(9) $$j=\underset{j}{argmin}\sqrt{{\left(v_{cx}^{\left(i\right)}-lp_{cx}^{\left(j\right)}\right)}^{2}+{\left(v_{cy}^{\left(i\right)}-lp_{cy}^{\left(j\right)}\right)}^{2}}.$$

It is because of the proposed geometric matching that the correspondence between the unordered vehicle set and the license plate set is obtained, making it possible to detect vehicles and license plates in parallel without additional detection adopted in VLN, thereby reducing model parameters as well as inference time.

### 2.4. Vehicle and License Plate Number Tracking

In order to facilitate the management of vehicles, their positions and license plate numbers are expected to be obtained at all times. However, the vehicles and license plates are inevitably obscured during their movements, illustrating the necessity of tracking. DeepSORT initializes a tracker to track a newly emerging vehicle and uses the Kalman filter to predict its position in the next frame. However, it only tracks vehicles without license plate numbers, making it an invalid predictor of the invisible license plate numbers.

An alternative solution is to improve DeepSORT with the adjacent frame (DeepSORTa). In this method, the obscured license plate number of the current frame is replaced and updated by the one in the adjacent frame. However, due to the ID switch and possible errors in license plate recognition, the prediction of obscured license plate numbers may be inaccurate.

**Figure 5:**
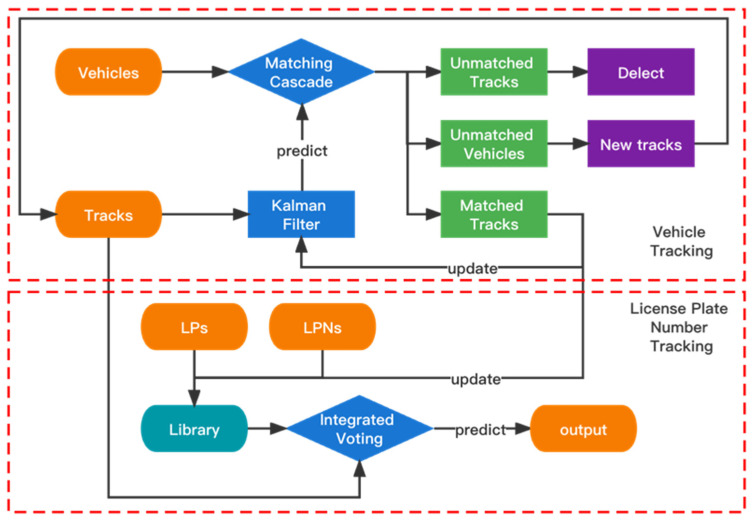
The structure of the proposed DeepSORTv.

Therefore, based on DeepSORT, this paper proposes DeepSORT with integrated voting (DeepSORTv). As shown in Figure 5, it is composed of the vehicle tracking and the license plate number tracking, both of which include update and prediction steps. The core difference is that for update and prediction, the vehicle tracking uses the Kalman filter, while the license plate number tracking uses integrated voting.

The details of DeepSORTv are as follows:

The vehicle bounding box $v^{\left(i\right)}$, the license plate bounding box $lp^{\left(i\right)}$, and the license plate number $lpn^{\left(i\right)}$, whose probabilities are, respectively, $P_{v}^{\left(i\right)}$, $P_{lp}^{\left(i\right)},\;\mathrm{and}\;P_{lpn}^{\left(i\right)}$, are obtained by PDM in Section 2.3. Among them, $P_{v}^{\left(i\right)}$ denotes the probability that bounding box $v^{\left(i\right)}$ contains a vehicle, $P_{lp}^{\left(i\right)}$ denotes the probability that bounding box $lp^{\left(i\right)}$ contains a license plate, and $P_{lpn}^{\left(i\right)}$ denotes the probability that the predicted $lpn^{\left(i\right)}$ is the correct license plate number of $lp^{\left(i\right)}$.

In the vehicle tracking, a tracker is created to track a vehicle, and each tracker forms a vehicle trajectory. According to the vehicle position at time t − 1, the Kalman filter is used to predict its position at time t. Next, the detected vehicle bounding box at time t is input as an observation. Then, the predictions and observations are matched according to the Matching Cascade based on the Mahalanobis distance, cosine distance, and IOU distance, as detailed in DeepSORT [18].

There are three possibilities for matching: if the observation cannot match any prediction, it indicates that the vehicle is newly detected, then a new tracker will be created to track it; if the prediction cannot match any observation, it indicates that the vehicle has left, then the tracker that makes this prediction will be deleted; if the observation and the prediction are matched, it indicates that the vehicle has been tracked before, then the prediction will be rectified and the parameters of the Kalman filter will be updated by the observation.

The license plate number tracking includes two steps: update step and prediction step. Their details are as follows:

Update step: If the observation and the prediction are matched, it indicates that the vehicle $v^{\left(i\right)}$ has been tracked by the tracker $T^{\left(k\right)}$. The license plate $lp^{\left(i\right)}$ and the license plate number $lpn^{\left(i\right)}$ of the vehicle $v^{\left(i\right)}$ will be added to the license plate library in the form of $record=T^{\left(k\right)}:\left(lp^{\left(i\right)},P_{lp}^{\left(i\right)},lpn^{\left(i\right)},P_{lpn}^{\left(i\right)}\right)$.

Prediction step: Traverse each tracker $T^{\left(k\right)}\left(k=1,2,3,\ldots ,K\right)$ and take out all the license plate records of $T^{\left(k\right)}$ in the library. Each record represents a voting message. The voted object is $lpn^{\left(i\right)}$, whose weight is $P_{lp}^{\left(i\right)}\times P_{lpn}^{\left(i\right)}$. The voting weights of the same license plate number are accumulated, and the final license plate number of tracker $T^{\left(k\right)}$ at time t ($\mathrm{lp}n_{t}$) is the one with the most votes. The calculation formula is as follows:

(10) $$lpn_{t}=\underset{T^{\left(k\right)}}{\arg\max}\sum^{\;}(P_{lp}\times P_{lpn}).$$

### 2.5. Entry and Exit Judgment

In terms of vehicle management, accurate vehicle entry–exit time and license plate numbers are required. An available way is to utilize the adjacent state detector (ASD) to judge the vehicle behavior of entry or exit according to adjacent frame images. It divides the area that is in the view of the camera into a parking area and a non-parking area. When the vehicle drives from the non-parking area into the parking area, it is judged to be the behavior of entry. On the contrary, it is judged to be the behavior of exit. However, this way only depends on adjacent frame images, so it is sensitive to data perturbation, leading to frequent misjudgments.

Therefore, this paper proposes the cumulative state detector (CSD) to judge the vehicle behavior of entry or exit. The details are as follows: Assume the vehicle v at time t is $v_{t}$ and its state is $S\left(v_{t}\right)$. $S\left(v_{t}\right)$ is calculated as

(11) $$S\left(v_{t}\right)=\{\begin{array}{c} 1,\;v_{t}\;\mathrm{in}\;a\;\mathrm{parking}\;\mathrm{area}\\ -1,\;v_{t}\;\mathrm{not}\;\mathrm{in}\;a\;\mathrm{parking}\;\mathrm{area} \end{array}$$

The cumulative state f(v,T) of vehicle v from time 0 to T is

(12) $$f\left(v,T\right)=\{\begin{array}{c} L,\overset{T}{\underset{t=0}{\sum }}S\left(v_{t}\right)>L\\ \overset{T}{\underset{t=0}{\sum }}S\left(v_{t}\right),-L\leq \overset{T}{\underset{t=0}{\sum }}S\left(v_{t}\right)\leq \;L\\ -L,\overset{T}{\underset{t=0}{\sum }}S\left(v_{t}\right)<-L \end{array}.$$

When the vehicle is in the parking area, its state is 1, at which time the possibility of entry behavior increases and f(v,T) increases. When f(v,T) increases to L, the entry behavior is completed and f(v,T) remains unchanged. When the vehicle is in the non-parking area, its state is −1. At this time, the possibility of exit behavior increases and f(v,T) decreases. When f(v,T) decreases to −L, the exit behavior is completed and f(v,T) remains unchanged.

In this paper, $\frac{L}{2}$ and $-\frac{L}{2}$ are the judgment points of entry and exit, respectively. When f(v,T) increases to $\frac{L}{2}$, T is the entry time; when f(v,T) decreases to $-\frac{L}{2}$, T is the exit time.

## 3. Datasets

Existing datasets such as VOC [26], COCO [27], and MOT [28,29,30] can be utilized to evaluate the performance of detection and tracking algorithms, but none of them are specialized for on-street parking vehicle scenes. Therefore, in order to better evaluate the proposed algorithm, eight cameras are installed in on-street parking lots in Ningbo, Zhejiang Province, China. The model number of those cameras is SNT-SC800 and the frames per second (FPS) is 25.

The data of on-street parking vehicles are collected and annotated to form three datasets: (1) The vehicle detection dataset: evaluate the precision of the detection algorithms. (2) The vehicle tracking dataset: evaluate the accuracy of the vehicle trajectories. (3) The vehicle entry–exit dataset: evaluate the time precision and the license plate number accuracy of vehicle entry and exit.

### 3.1. Vehicle Detection Dataset

The attributes of the dataset are shown in Table 1, which contains 4768 images with two categories, the vehicle and the license plate. The instance number of vehicles and license plates are, respectively, 16,291 and 11,030.

**Figure 6:**
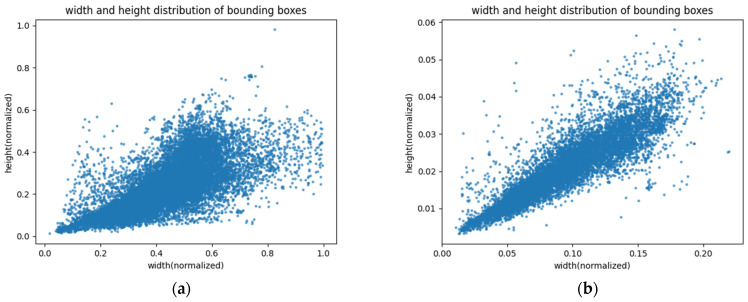
The size distribution of the normalized ground truth bounding boxes. (**a**) The size distribution for vehicles; (**b**) The size distribution for license plates.

Figure 6 shows the size distribution of the normalized ground truth bounding boxes, from which it can be seen that our dataset has a relatively uniform distribution of object sizes.

**Figure 7:**
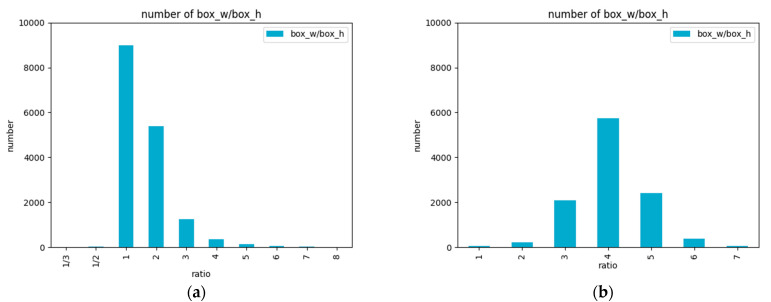
The number of aspect ratios of the ground truth bounding boxes. (**a**) The number of aspect ratios for vehicles; (**b**) The number of aspect ratios for license plates.

The number of aspect ratios for the ground truth bounding boxes is shown in Figure 7. It presents that the ratio of the vehicles is concentrated in 1 and 2, and the ratio of the license plates is concentrated in 3, 4, and 5.

### 3.2. Vehicle Tracking Dataset

The dataset includes 16 vehicle videos, 8 for daytime scenes and 8 for nighttime scenes. Each video is labeled frame by frame and applied to evaluate the performance of the tracking algorithm. As in Table 2, the number of frames per video varies from 550 to 2545. Every video contains 4–19 IDs, each of which represents a trajectory with a minimum of 19 frames and a maximum of 2545 frames.

### 3.3. Vehicle Entry–Exit Dataset

A total of 156 videos are captured to record vehicle entry and exit, including 78 entry and 78 exit videos, respectively. Each video is labeled with (lpn, t), where lpn is the license plate number of vehicle entry or exit, and t is the time of its entry or exit.

This paper applies the precision curve to evaluate the precision of entry and exit time. Assume that the real time is $t_{r}$ and the predicted time is $t_{p}$. Given a time error threshold θ, the predicted time is correct when $\left|t_{p}-t_{r}\right|\leq \theta$ and incorrect when $\left|t_{p}-t_{r}\right|>\theta$. Each θ corresponds to a precision, and the precision curve is obtained by assigning different values to θ.

## 4. Experimental Results and Analysis

This paper evaluates the proposed algorithm on the vehicle detection dataset, the vehicle tracking dataset, and the vehicle entry–exit dataset. Our algorithm is implemented in Python using Pytorch [31], and runs with 32 cores of a 2.10 GHz Intel Xeon CPU E5-2620 and an NVIDIA TITAN XP GPU.

### 4.1. Detection Results

The detection precision of our proposed algorithm was evaluated based on the vehicle detection dataset. The input image is scaled to 640×640 and then input to the PDM. The evaluation is carried out according to the following metrics:

- Precision: The proportion of correct predictions in the detected bounding boxes.
- Recall: The proportion of correct predictions in the ground truth bounding boxes.
- Average Precision (AP): The area under the Precision–Recall curve.
- Mean Average Precision (mAP): The average value of AP for all categories.

**Figure 8:**
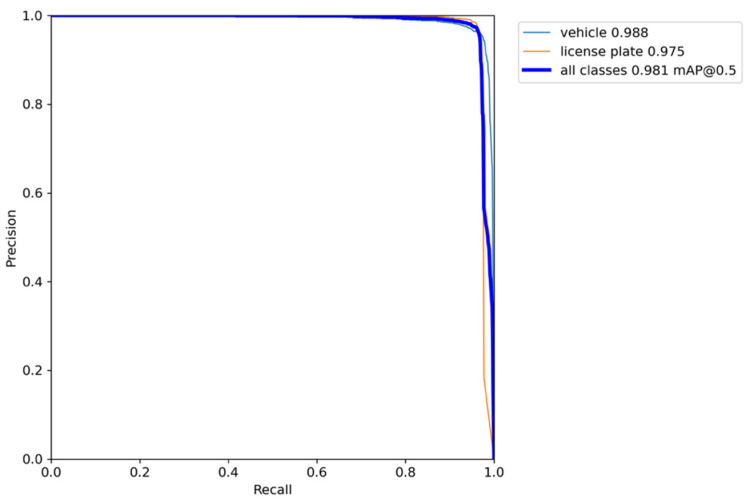
The precision–recall curve of the detection results.

The detection results are shown in Table 3 and the precision–recall curve is presented in Figure 8. In our algorithm, the mAP@0.5 is 98.1, the AP@0.5 of the vehicle is 98.8, and the AP@0.5 of the license plate is 97.5. It reveals that our algorithm can detect the objects of all categories with high precision and recall. In particular, from Figure 6b, it can be calculated that the widths of the license plates are 0–128 pixels and the heights are 0–38.4 pixels. For such small objects, they can still have a recall of 96.2 and a precision of 98.2, which reflects that our algorithm has high precision on small objects detection.

As shown in Table 4, compared to the VLN, our algorithm has the following advantages:

In terms of the inference speed, our algorithm takes only 20.4 ms to infer an image, which is close to 1/5 of the time required by the VLN. The reason is that instead of detecting the vehicles first and then the license plates, our algorithm makes a parallel detection of all the vehicles and license plates in the image all at once and then matches them based on their geometric relationships, which greatly reduces the inference time.

In terms of the model size, the number of parameters in our model is only half that of the VLN. The reason is that instead of detecting the vehicles and the license plates with two separate models, our algorithm shares the same feature extraction network, significantly reducing the model parameters.

### 4.2. Tracking Results

The vehicle tracking dataset and the vehicle entry–exit dataset are applied to evaluate the tracking performance of our proposed algorithm. The following metrics are applied in the evaluation:

- MOTA: Multi-object tracking accuracy.
- MOTP: Multi-object tracking precision.
- MT: Number of mostly tracked trajectories.
- ML: Number of mostly lost trajectories.
- FP: The total number of false positives.
- FN: The total number of false negatives.
- ID switch: The times a trajectory changes.
- VLPA: The vehicle entry–exit license plate number accuracy.

The tracking results are shown in Table 5. The total number of ID switches is only 16, with an average of only 1 per video, indicating that our algorithm is able to identify different vehicles excellently. MOTA is the overall tracking accuracy calculated from FP, FN, and ID switch. MOTP reflects the matching degree of the predicted bounding box and the ground truth bounding box. The MOTA of our algorithm is 92.1% and the MOTP is 89.4%, demonstrating the good performance of our algorithm.

As presented in Table 6, the license plate number tracking accuracy of our proposed DeepSORTv is 92.95%, which is 42.31 percentage points higher than DeepSORT and 23.08 higher than DeepSORTa. This is because, in our algorithm, the current license plate number is obtained by integrated voting based on the historical license plate library, in which two advantages exist: (1) The tracking variance is reduced compared to using only a single frame or adjacent frames. (2) If the license plate is obscured, our algorithm can accurately infer the current license plate number.

### 4.3. Entry and Exit Results

**Figure 9:**
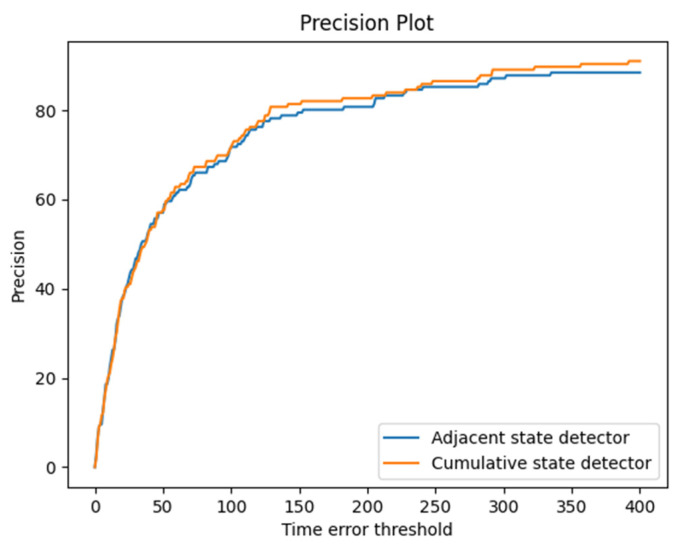
The precision curves of different state detectors.

The vehicle entry–exit dataset is applied to evaluate the precision of vehicle entry and exit time. Figure 9 illustrates the precision curves of the adjacent state detector and our proposed cumulative state detector. When the time error threshold is 0–50, the precision of the cumulative state detector is close to that of the adjacent state detector. When the time error threshold is greater than 50, the precision of the cumulative state detector is higher than that of the adjacent state detector. It indicates that our proposed cumulative state detector has higher precision. The reason is that when the predicted vehicle position is incorrect, the adjacent state detector judges the vehicle behavior of entry or exit according to the inaccurate vehicle information, while the cumulative state detector can correct it by subsequent vehicle positions.

In addition, as shown in Table 7, the accuracy of the cumulative state detector is also 6.41 percentage points higher than the adjacent state detector.

### 4.4. Qualitative Analysis

**Figure 10:**
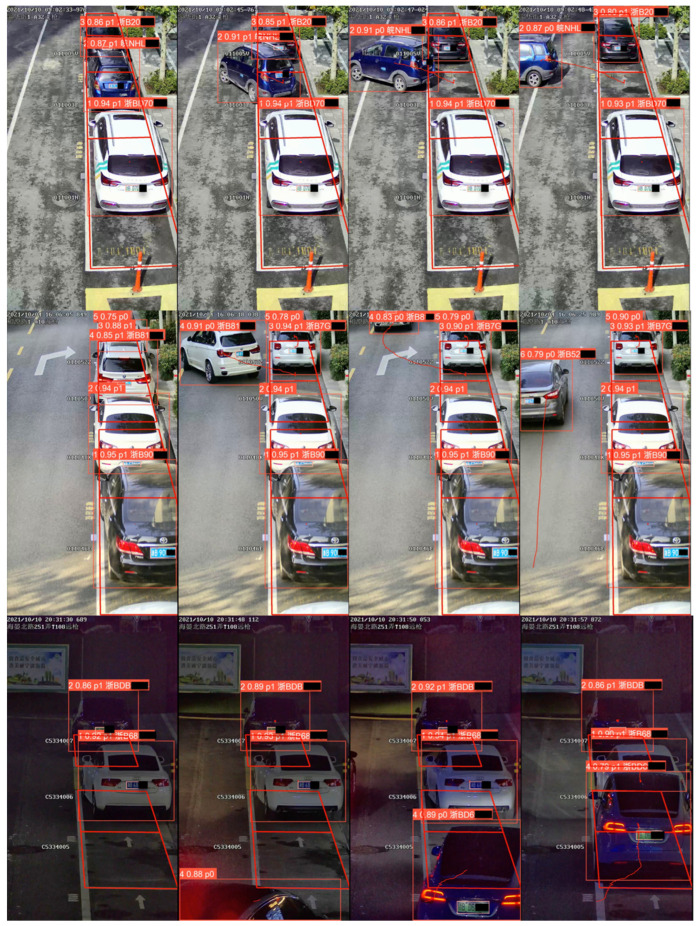
The qualitative results. (For privacy protection, license plate numbers are pixelated).

Figure 10 introduces the visualization results of our algorithm, in which each row represents a video’s detection and tracking result, and each column from left to right reflects the increase in the time.

Each bounding box is visualized as the label (Vehicle ID, Probability, Parking flag, License plate number), whose specific meanings are as follows:

- Vehicle ID: The ID of each vehicle is different. Under ideal circumstances, it remains unchanged throughout the tracking process.
- Probability: The probability that the predicted bounding box contains a vehicle.
- Parking flag: This is 1 when the vehicle is in the parking area, and 0 in the non-parking area.
- License plate number: This refers to the license plate number belonging to the predicted bounding box. If the license plate number is unpredicted, the corresponding part of the label is kept as empty.

In addition, the trajectories of every vehicle are plotted in the videos.

It can be seen from Figure 10 that the predicted bounding boxes are of high precision and the license plate numbers are of remarkable accuracy. In addition, the correspondences between the vehicles and the license plates are also accurate. In the aspect of tracking, the vehicle IDs remain unchanged and the vehicle trajectories are smooth. Furthermore, even if the license plates of the vehicles are not visible, our algorithm can still accurately predict their license plate numbers. For instance, in row 1 column 4, the license plate of the vehicle (ID 2) is not visible. In row 3 column 4, the license plate of the vehicle (ID 1) is completely obscured by the vehicle (ID 4). However, they can also be predicted by our proposed algorithm. The above reflects the significant efficiency of our algorithm.

## 5. Limitations

This paper only considers video detection and tracking for a single camera, causing the limitation in scenes with a large range of view.

## 6. Conclusions and Future Work

This paper proposes an open-space vehicle positioning algorithm based on detection and tracking in video sequences. The experimental study is conducted to explore the precision and speed of detection, the performance of tracking, as well as the precision of the entry and exit time.

Our work is as follows: (1) Carry out parallel detection of vehicles and license plates, and then match them based on their geometric relationships. (2) Improve DeepSORT by combining with integrated voting to infer vehicles’ positions and license plate numbers. (3) Judge the vehicle behavior of entry or exit by the cumulative state detector to increase the fault-tolerance.

The experimental results of the above work reveal that our algorithm has fewer parameters, faster inference speed, higher precision, and more accurate tracking, demonstrating that our algorithm can be well applied to open-space vehicle positioning. However, this paper only considers video detection and tracking for a single camera, which has a limited field of view. In the future work, the problem of multi-camera video detection and tracking will be focused, including multi-camera detection, cross-camera tracking, and multi-camera collaboration.

## Figures and Tables

**Table 1:** The attributes of the vehicle detection dataset.

Attribute	Value
Total number of images	4768
Number of training images	4292
Number of validation images	476
Number of categories	2
Categories	vehicle, license plate
Instance number of vehicles	16,291
Instance number of license plates	11,030

**Table 2:** The attributes of the vehicle tracking dataset.

Attribute	Value
Total number of videos	16
Number of videos for daytime scenes	8
Number of videos for nighttime scenes	8
Number of frames per video	550–2545, Avg. 1210
Number of IDs per video	4–19, Avg. 7.31
Number of frames per ID	19–2545, Avg. 799

**Table 3:** The detection results of the proposed algorithm.

Category	Precision	Recall	mAP@0.5	mAP@0.5:0.95
Vehicle	95.6	97.2	98.8	77.8
License plate	98.2	96.2	97.5	75.4
Overall	96.9	96.7	98.1	76.6

**Table 4:** The parameters and inference time of different detection modules.

Method	Input Network Resolution	Parameters	Inference Time (ms)
VLN	640×640	14,025,644	95.3
PDM (ours)	640×640	7,015,519	20.4

**Table 5:** The tracking results of the proposed algorithm.

Metrics	MOTA	MOTP	MT	ML	FP	FN	ID switch	VLPA
Results	92.1%	89.4%	63.1%	14.8%	816	6552	16	92.95%

**Table 6:** The VLPA of different tracking modules.

Method	VLPA
DeepSORT	50.64%
DeepSORTa	69.87%
DeepSORTv (ours)	92.95%

**Table 7:** The VLPA of different state detectors.

Method	VLPA
Adjacent state detector	86.54%
Cumulative state detector (ours)	92.95%

## Data Availability

Not applicable.
